# On the Effect of the Synthesis Route of the Support in Co_3_O_4_/CeO_2_ Catalysts for the Complete Oxidation of Methane

**DOI:** 10.1021/acs.iecr.2c03245

**Published:** 2022-12-06

**Authors:** Andoni Choya, Beatriz de Rivas, Jose I. Gutiérrez-Ortiz, Rubén López-Fonseca

**Affiliations:** Chemical Technologies for Environmental Sustainability Group, Department of Chemical Engineering, Faculty of Science and Technology, University of the Basque Country UPV/EHU, Barrio Sarriena s/n, Leioa, Bizkaia E-48940, Spain

## Abstract

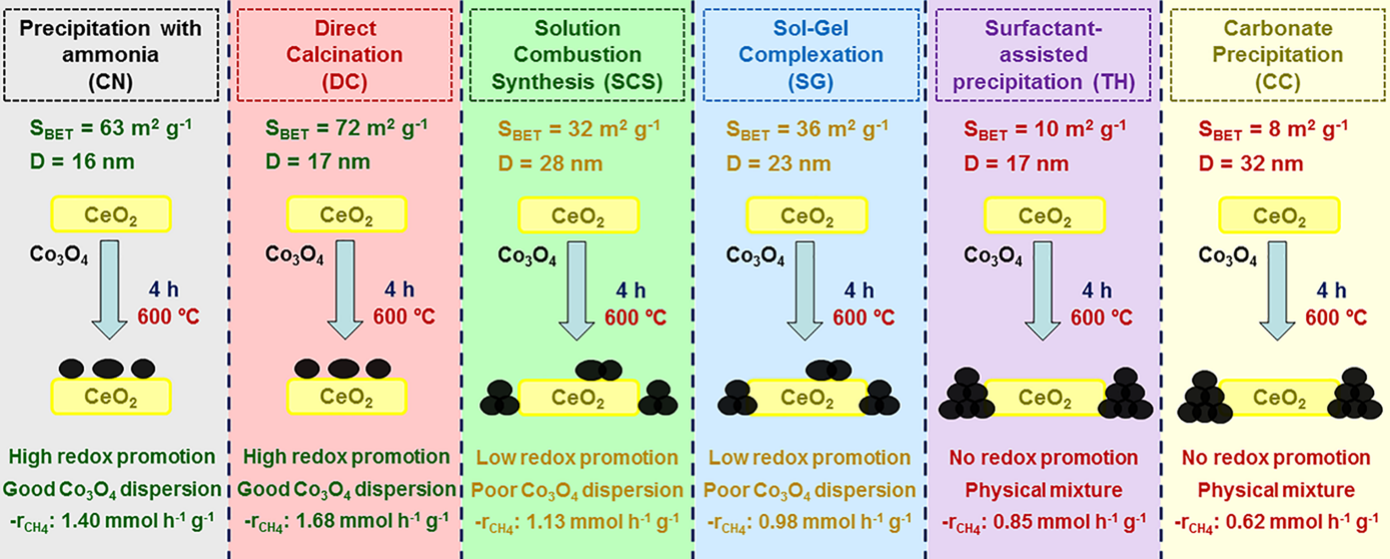

Six ceria supports synthesized by various synthesis methodologies were used to deposit cobalt oxide. The catalysts were thoroughly characterized, and their catalytic activity for complete methane oxidation was studied. The supports synthesized by direct calcination and precipitation with ammonia exhibited the best textural and structural properties as well as the highest degree of oxidation. The remaining supports presented poorer textural properties to be employed as catalytic supports. The cobalt deposited over the first two supports presented a good dispersion at the external surface, which induced a significant redox effect that increased the number of Co^3+^ ions on their surface. Consequently, the presence of highly active lattice oxygen species on the surface of these catalysts was favored. Additionally, the optimal active catalyst (Co-DC) revealed a significant resistance to water vapor inhibition, owing to the high hydrophobicity of the ceria support.

## 1 Introduction

In recent years, industrial development and population growth have brought with them several negative effects. In particular, climate change, produced by the extensive anthropogenic emission of greenhouse gases, is one that requires an immediate response from all sectors of society to minimize its potential damage to the planet and to humankind. For this reason, alternatives to reduce the emissions of these gases from human activities are being intensively sought. According to the European Environment Agency, the sector that makes the largest contribution to the emission of greenhouse gases is transport^1^ and, more specifically, road transport, with passenger cars and heavy-duty vehicles generating the majority of the associated emissions.^2^ Thus, promoting the use of alternative fuels with lower emissions is a promising strategy to decrease the environmental impact of transport. One of those alternative fuels is vehicular natural gas.

Vehicular natural gas (or VNG) is considered to be a reliable alternative to traditional liquid fuels, namely, gasoline and diesel, and can serve as a transition technology to H_2_-based energy sources. Vehicles fueled with VNG produce between 20 and 30% less CO_2_ and 50 and 80% less NO_*x*_ than traditional fuels, achieving reductions of up to 50–80%. In addition, they emit almost no sulfur oxide and virtually no particulate matter at all.^3−5^ However, the burning of natural gas is not complete in internal combustion engines owing to being a slow process. Therefore, there is a certain amount of residual methane in the exhaust gases from the engine that needs to be removed before venting them into the atmosphere due to the high global warming potential of methane. For this purpose, catalytic oxidation is the most commonly used technique.

The combustion of methane requires the activation of the C–H bonds in the molecule. Therefore, the selected catalysts are required to exhibit very high activity at relatively low temperatures, working with low concentrations of methane (below 1%), while also maintaining an excellent selectivity toward CO_2_. Moreover, the presence of concentrations of up to 10% water vapor and CO_2_ (and other pollutants in smaller concentrations) may induce an important deactivation effect on the chosen catalyst, limiting both its activity and lifetime.^6,7^

Spinel-type cobalt oxide (Co_3_O_4_) is widely known for being an active catalyst in environmental applications and has been extensively investigated for the oxidation of VOCs,^8^ the abatement of N_2_O, and the combustion of soot, among many other reactions.^9,10^ In all cases, the origin of its activity has been connected to its good redox properties, that is, its high reducibility at moderate temperatures. This is a consequence of the ease of alternating between the +3 and +2 oxidation states that the cobalt ions possess, thus providing high mobility to the oxygen species of the crystalline lattice.^11,12^ Accordingly, Co_3_O_4_ has been also studied for the oxidation of methane,^13,14^ especially under lean conditions, where the catalysts based on noble metals, such as platinum and palladium, have always been the preferred option.^15,16^

However, when Co_3_O_4_ is used in its bulk form it generally presents poor textural and structural properties, especially if it is prepared by conventional synthesis methodologies such as sol–gel or precipitation.^15,17^ For this reason, Co_3_O_4_ is usually deposited over the surface of a porous support as a way to improve its physicochemical characteristics. The most commonly employed supports for cobalt oxide are alumina,^18^ ceria,^19^ zirconia,^20^ silica,^21^ magnesia,^22^ zeolites, and cordierite.^23,24^ Each of them has a different effect on the properties of the supported cobalt catalyst due to the varying nature of its interaction with cobalt oxide. For instance, some supports, namely, alumina, silica, and magnesia, are generally highly porous and greatly improve the textural and structural properties of the deposited cobalt. However, their strong metal oxide–support interaction tends to be detrimental due to the formation of inactive phases such as cobalt aluminate, Co–Mg mixed oxides, and cobalt silicate.^25,26^ This negative effect may hide any activity enhancement eventually derived from the improvement of the textural properties.

Interestingly, supports such as ceria can induce a beneficial promotion of the redox behavior of the Co_3_O_4_ owing to its storage and mobility of oxygen species. Indeed, it is the most commonly employed promoter when it comes to redox promotion. However, these materials tend to present poorer textural properties with respect to alumina and silica,^27,28^ and obtaining them in a high-surface form usually requires complex synthesis methodologies.^29,30^ In addition, the chosen synthesis methodology generally strongly affects the physicochemical properties of the obtained ceria. The most relevant factors are the thermal activation, the addition of surfactants and other structural promoters, and the synthesis temperature.^31,32^

According to the literature, ceria can be synthesized, with textural properties good enough to be used as a support, by various routes such as precipitation,^33^ solution combustion,^34^ sol–gel,^35^ hydrothermal synthesis,^36^ and mechanochemical synthesis.^37^ Many of these synthesis methodologies have been used to prepare ceria supports for cobalt oxide catalysts. However, the differences in the subsequent strategy for cobalt deposition and the employed reaction conditions make it difficult to assess the influence of the synthesis methodology of the ceria support on the properties of each of the resulting Co_3_O_4_/CeO_2_ catalysts.

Hence, in this work we systematically studied the influence of the synthesis methodology of the ceria support on the physicochemical properties of Co_3_O_4_/CeO_2_ catalysts and their activity for the complete oxidation of methane. For this purpose, six different ceria supports were synthesized by various routes and subsequently employed as supports to deposit Co_3_O_4_. The resulting supports and catalysts were thoroughly characterized, and the efficiency of the cobalt catalysts was analyzed under realistic conditions.

## 2 Experimental Methods

### 2.1 Synthesis of the CeO_2_ Supports and Co_3_O_4_/CeO_2_ Catalysts

Three of the six CeO_2_ supports were prepared following precipitation routes. The first one, denoted as CN, was obtained by the precipitation of a cerium nitrate hexahydrate (Ce(NO_3_)_3_·6H_2_O) solution at 80 °C with an aqueous solution of 3 M ammonium hydroxide until pH 10.^38^ The second one, denoted as CC, used a 1.2 M solution of sodium carbonate as the precipitating agent, which was added until pH 8.5.^39^ Finally, the third precipitation route, denoted as TH, followed the so-called Taguchi method. In this route, the precipitation was carried out until pH 12 with a 0.5 M sodium hydroxide solution and cetyltrimethylammonium bromide was added to promote the structure of the precipitated ceria precursor.^40^ In these three synthesis routes, the precipitation step lasted 30 min, to which was added another 30 min of aging time. After the aging step, the precipitates were filtered under vacuum and thoroughly washed with at least 8 L of deionized water to remove residual sodium ions.

Another ceria support was obtained by direct calcination, denoted as DC, which simply involved the calcination of the cerium precursor at high temperature (600 °C for 4 h). A fifth support, denoted as SCS, was prepared by solution combustion synthesis using the same cerium precursor and glycine (C_2_H_5_NO_2_) as the fuel in stoichiometric proportions.^41^ Once the solution was prepared, batches of 0.5 cm^3^ were poured into ceramic crucibles and placed inside an oven at 300 °C for 30 min to initiate the combustion reaction. Finally, the last ceria support, denoted as SG, was synthesized by sol–gel complexation, also known as the citrate method. Hence, a solution of cerium nitrate and citric acid was evaporated until it formed a gel that was further dried at 110 °C, resulting in a highly porous solid.^42^

The Co_3_O_4_/CeO_2_ supported catalysts were prepared by depositing a cobalt precursor over the previously synthesized ceria supports by the basic precipitation method. The selected nominal Co content was 30 wt % .Thus, cobalt nitrate hexahydrate (Co(NO_3_)_2_·6H_2_O) was used as the cobalt precursor and sodium carbonate was used as the precipitating agent, which was added dropwise until pH 8.5 at 80 °C. All synthesized CeO_2_ supports and Co_3_O_4_/CeO_2_ catalysts were subjected to calcination at 600 °C for 4 h to obtain their respective final forms.

### 2.2 Characterization Techniques

The N_2_ adsorption/desorption isotherms of the supports and catalysts were obtained with a Micromeritics Tristar II apparatus at 77 K. Prior to the analysis, each sample was subjected to degassing at 300 °C for 10 h in a Micromeritics SmartPrep degasser. The specific surface area of the samples was calculated from the adsorption isotherm using the BET method, while the pore volume and pore size distributions were determined from the desorption experiment using the BJH method.

The elemental composition of the cobalt catalysts was determined by ICP-AES in a SPECTRO Spectrogreen DSOI optimal emission spectrometer. For the analysis, 5 to 6 mg of each sample was subjected to acid digestion, in triplicate, in an SCP SCIENCE DigiPREP Jr digestion block and then diluted into 50 cm^3^ of Milli-Q water.

X-ray diffraction experiments were carried out on an X’PERT-PRO X-ray diffractometer equipped with a Cu Kα (λ = 1.5406 Å) X-ray source that was operated at 40 kV and 40 mA and a Ni filter. The diffractograms were taken between the 2θ positions of 5 and 80° with a step size of 0.026°. Elemental maps of the cobalt supports were obtained with a Cs-corrected Thermofisher Scientific Titan STEM microscope operated at 300 kV. The microscope was equipped with a 2k × 2k Ultrascan CCD Gatan camera, a HAADF Fischione detector, and an Ultim Max detector for energy dispersive X-ray spectroscopy (EDS). XPS spectra were recorded with a Kratos AXIS Supra spectrometer using a 225 W Al Kα radiation source with a pass energy of 20 eV. The registered spectra were corrected by fixing the signal of adventitious carbon at a binding energy of 284.6 eV, and a Shirley-type background was used for the deconvolution of the spectra.

The reducibility and reactivity of the oxygen species of the supports and catalysts were investigated on a Micromeritics Autochem 2920 apparatus by temperature-programmed techniques. On one hand, H_2_-TPR experiments were carried out using a 5% H_2_/Ar mixture as the reducing agent from ambient temperature to 900 °C. On the other hand, for the CH_4_-TPRe experiments, a 5% CH_4_/He mixture was used, and the experiments were carried out from ambient temperature to 600 °C. The reaction products from CH_4_-TPRe runs were monitored with a MKS Cirrus quadrupole mass spectrometer.

### 2.3 Catalytic Activity and Stability Testing

The activity and stability of the synthesized catalysts was assessed in a PID Eng&Tech Microactivity Reference bench-scale fixed-bed tubular reactor. In each experiment, 1 g of catalyst (0.25–0.3 mm particles) were diluted with 1 g of quartz (0.5–0.8 mm particles) and placed inside a stainless steel tube with a type-K thermocouple placed inside to control the temperature of the catalytic bed. The light-off experiments were carried out from 200 to 600 °C by feeding a gaseous mixture of 1%CH_4_/10%O_2_/89%N_2_ at 500 cm^3^ min^–1^. Under these conditions, the GHSV was around 60 000 h^–1^.

In addition, stability tests during a prolonged time on stream were carried out at a constant temperature of 525 °C. The gaseous mixture fed to the reactor was changed every 25 h during the total 150 h of experiment. The test started with the previously described feedstream, and after 25 h, CO_2_ was added. Then, it followed a second 25 h period of the initial feedstream, and after that, 10% vol. water vapor was fed by a liquid pump through an evaporator that was kept at 150 °C to ensure its complete vaporization. Finally, after a third 25 h period with the initial feedstream (CH_4_/O_2_), 10% CO_2_ and 10 vol % water vapor were coadded for the last 25 h of experiment. The analysis of the outlet stream from the reactor was carried out with an Agilent Technologies MicroGC equipped with a TCD. The methane conversion was calculated by measuring the differences in concentration between the inlet and outlet streams.

## 3 Results and Discussion

### 3.1 Physicochemical Characterization of the CeO_2_ Supports

The selected synthesis methodologies produced six different CeO_2_ supports that were characterized by N_2_ physisorption, XRD, XPS, and H_2_-TPR in order to analyze their textural and structural properties, surface composition, and redox behavior. Table 1 lists the specific surface area and pore volume of the samples. The corresponding isotherms are included in Figure S1, Supporting Information and suggest that the as-prepared samples are mesoporous materials (type IV isotherms with an H1 hysteresis cycle).^43^ Remarkable differences in textural properties were found as a function of the synthesis route. As for the BET surface area, the values varied from 8 to 72 m^2^ g^–1^. Thus, the samples could be ranked as relatively highly porous (in the case of the DC and CN samples with values in the 63–72 m^2^ g^–1^ range), nonporous (in the case of the CC and TH oxides, 8–10 m^2^ g^–1^), and moderately porous (in the case of the SCS and SG samples, 32–36 m^2^ g^–1^). Simultaneously, three different pore volumes were estimated: 0.03 (CC and TH), 0.08 (SCS and SG), and 0.13–0.15 cm^3^ g^–1^ (DC and CN).

**Table 1:** Textural and Structural Properties of the Synthesized CeO_2_ Supports

support	BET surface area, m^2^ g^–1^	pore volume, cm^3^ g^–1^	CeO_2_ crystallite size, nm
CC	8	0.03	32
CN	63	0.13	16
DC	72	0.15	17
SCS	32	0.08	28
SG	36	0.08	23
TH	10	0.03	17

Following comparable behavior, the pore size distributions of the supports (Figure S2, Supporting Information), obtained by the BJH method, showed a high variability. Hence, the oxides obtained by the CN and DC routes presented comparable bimodal distributions with a large maximum at around 75–90 Å and a smaller maximum at 25 Å. Similarly, the SCS and SG supports presented a maximum at around 75–90 Å, although the latter also exhibited a narrow maximum at 35 Å. By contrast, the TH and CC supports exhibited unimodal distributions with very different maxima. Thus, the former exhibited only small pores of around 20 Å while the latter showed only mesopores of around 300 Å.

The results of the structural characterization of the supports by XRD are shown in Figure S3, Supporting Information. All of the prepared samples exhibited diffraction signals at 2θ = 28.6, 33.1, 47.5, 56.3, 59.1, 69.4, 76.7, and 79.1°, attributable to a cubic phase of ceria (ICCD 00-004-0593). No signals assignable to any reduced phase (such as Ce_2_O_3_) were detected. The average crystallite size, estimated by the Scherrer equation, is shown in Table 1. Thus, the CN, DC, and TH samples presented a crystallite size of around 17 nm, while for the other three methodologies (CC, SCS, and SG) the crystallite size was larger, between 23 and 32 nm.

The surface structure of the synthesized CeO_2_ supports was investigated by XPS. The Ce 3d and O 1s XPS spectra of the CeO_2_ supports are shown in Figure S4, Supporting Information. The Ce 3d spectra were deconvoluted into 10 peaks, which, following the convention adopted by Romeo et al.,^44^ were identified with the letters U and V to refer to the 3d_5/2_ and 3d_3/2_ spin–orbit components, respectively. From the five pairs of peaks, those named V, U; V’’, U’’; and V’’’, U’’’ were associated with the presence of Ce^4+^ cations, while the remaining pairs (V_0_, U_0_ and V’, U’) were attributed to the Ce^3+^ species. On the other hand, the O 1s spectra were fitted with three bands, located at 528.4, 530.5, and 533.6 eV. The first signal was attributed to the oxygen species from the CeO_2_ lattice, the second signal was associated with the oxygen species weakly adsorbed on the surface, and the last signal was related to the presence of hydroxyl species, carbonates, and water molecules.^45,46^

The surface composition in terms of Ce and O weight percentages and the Ce^4+^/Ce^3+^ and O_latt_/O_ads_ molar ratios was estimated from the spectral deconvolution. The results are summarized in Table 2. The amount of cerium in the CeO_2_ supports varied significantly, between 81.1 wt % for the DC sample and 73.3 wt % for the TH support. Since the theoretical weight percentage of cerium in pure CeO_2_ is 81.4 wt %, these results evidenced that all synthesized oxides presented defects on their surfaces. The oxides with the lowest abundance of cerium (TH, CC, and SG) had the largest number of defects. The Ce^4+^/Ce^3+^ molar ratio of the oxides was in line with the previous finding, since the highly defective supports (DC, CN, and SCS) evidenced the highest Ce^4+^/Ce^3+^ molar ratio (2.46–2.69) and the largest abundance of oxygen lattice species (2.29–2.63).

**Table 2:** Surface Composition of the Synthesized CeO_2_ Supports

support	Ce, wt %	O, wt %	Ce^4+^/Ce^3+^ molar ratio	O_latt_/O_ads_ molar ratio
CC	76.6	23.4	2.19	1.57
CN	80.5	19.5	2.49	2.54
DC	81.1	18.9	2.64	2.63
SCS	80.1	20.9	2.46	2.29
SG	79.1	19.9	1.96	1.81
TH	73.3	26.3	2.02	1.66

**Figure 1:**
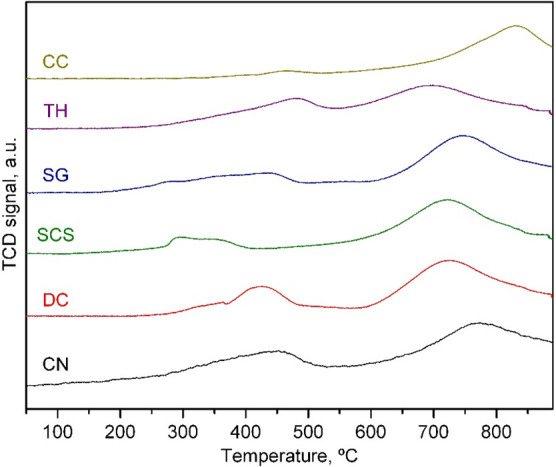
TPR profiles of the CeO_2_ supports.

Finally, the redox properties of the prepared CeO_2_ supports were studied by temperature-programmed reduction with H_2_ (H_2_-TPR). The TPR profiles of all of the samples, which are included in Figure 1, exhibited the typical two-step reduction process of ceria.^47^ Following this process, the first reduction peak, centered between 300 and 450 °C, was assigned to the reduction of the surface of the oxide, while the second reduction peak, located between 700 and 850 °C, was associated with the reduction of the bulk of the CeO_2_. However, among the various supports, the peak reduction temperature of each step varied significantly. Thus, the CN, SCS, and SG oxides required the lowest temperatures for the first reduction step. As for the reduction of the bulk, the TH, DC, and SCS oxides evidenced better redox behavior with peak temperatures of between 675 and 700 °C. The integration and quantification of the TPR traces allowed the calculation of the H_2_ uptake related to both reduction steps and the degree of reduction (taking as reference the theoretical H_2_ uptake associated with the reduction of CeO_2_ to Ce_2_O_3_, namely, 2.88 mmol H_2_ g^–1^). These results are included in Table 3. The most reducible CeO_2_ samples were those prepared by the CN and DC routes (degree of reduction of 54%), followed by the sample obtained by the TH route (degree of reduction of 51%). However, the sources of the reducibility of these three supports were not the same. It was found that for the former the reducibility of the bulk of CeO_2_ was markedly higher than that for the latter.

**Table 3:** H_2_ Uptakes and Degrees of Reduction of the Synthesized CeO_2_ Supports

support	low-temperature H_2_ uptake, mmol g^–1^	high-temperature H_2_ uptake, mmol g^–1^	total H_2_ uptake, mmol g^–1^	degree of reduction, %
CC	0.09	1.12	1.21	42
CN	0.39	1.18	1.57	54
DC	0.36	1.20	1.56	54
SCS	0.24	1.14	1.38	48
SG	0.36	1.01	1.37	47
TH	0.46	1.01	1.47	51

### 3.2 Physicochemical Characterization of the Co_3_O_4_/CeO_2_ Catalysts

The characterization of the synthesized cobalt catalysts was carried out with several techniques, namely, N_2_ physisorption, ICP-AES, XRD, STEM/EDS-HAADF, XPS, H_2_-TPR, and CH_4_-TPRe. As for the elemental composition of the supported catalysts, it must be stated that all of the samples had virtually identical Co content that was coincident with the nominal composition (30 wt % Co). The N_2_ physisorption isotherms of the Co_3_O_4_/CeO_2_ samples (Figure S5, Supporting Information) were identified in a similar way to those of the CeO_2_ supports (type IV and the H1 hysteresis cycle). The specific surface area and pore volume of the catalysts are summarized in Table 4. According to the difference in the specific surface area between the catalysts and their corresponding supports, three distinct behaviors could be discriminated. On one hand, the Co-CN and the Co-DC catalysts presented lower specific surface areas than their corresponding supports. On the other hand, Co-CC and Co-TH experienced an increase in their specific surface areas with respect to their respective ceria supports. Finally, the two remaining samples (Co-SCS and Co-SG) did not exhibit a significant variation from the specific surface areas of their corresponding supports.

**Table 4:** Textural and Structural Properties of the Synthesized Co_3_O_4_/CeO_2_ Catalysts^a^

catalyst	Co, wt %	BET surface area, m^2^ g^–1^	pore volume, cm^3^ g^–1^	Co_3_O_4_ crystallite size, nm	CeO_2_ crystallite size, nm
Co-CC	29.0	18 (8)	0.07	44	33
Co-CN	30.1	44 (63)	0.12	31	17
Co-DC	30.3	52 (72)	0.13	26	17
Co-SCS	30.9	33 (32)	0.11	34	25
Co-SG	30.9	39 (36)	0.12	41	23
Co-TH	30.5	36 (10)	0.10	37	17

a Values in parentheses correspond to the bare ceria supports.

This behavior could be better rationalized by comparing the pore size distributions of supports and catalysts, as shown in Figure S2, Supporting Information. Thus, the Co-CN and Co-DC catalysts presented bimodal distributions with one of the maxima centered in the same position as the maximum of their corresponding support (85 and 75 Å, respectively), but with a lower pore volume. This pointed out the reduction in the number of open pores due to plugging by deposited Co_3_O_4_ crystallites with sizes larger than the pore diameter. The second maximum was located at 35 Å for all catalysts and was associated with the small mesopores of the deposited Co_3_O_4_. Notice that the Co-DC catalyst presented the largest number of these mesopores. Conversely, the pore size distributions of the Co-TH and Co-CC catalysts presented a higher pore volume than did their corresponding ceria supports. This, along with the increase in the specific surface area, suggested that there was a significant amount of segregated Co_3_O_4_ in these two catalysts. More specifically, given the low pore volume of the TH support, the pore size distribution of the Co-TH catalyst could be assigned to that of the deposited Co_3_O_4_. Thus, the Co_3_O_4_ deposited in all catalysts would present a bimodal distribution with maxima located at 90 and 35 Å, in line with the previous results. Finally, the Co-SG and Co-SCS catalysts maintained their respective specific surface areas and pore volumes. It was believed that the reduction in the pore volume due to pore plugging in the 45–90 Å range was compensated by the increase in the number of pores of size around 35 Å, owing to the deposited Co_3_O_4_.

**Figure 2:**
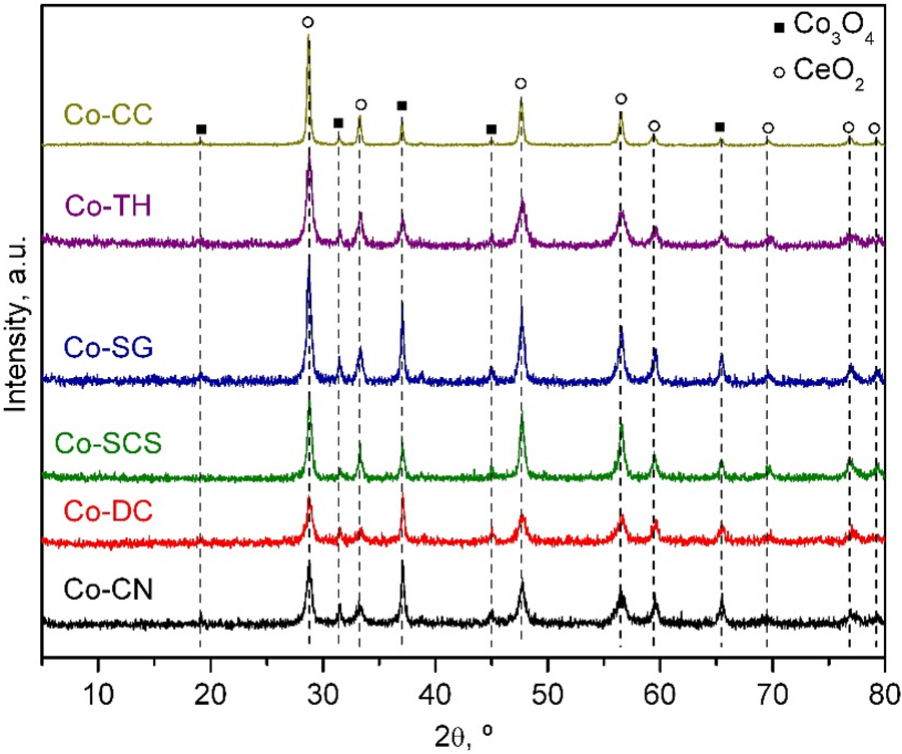
X-ray diffractograms of the Co_3_O_4_/CeO_2_ catalysts.

The X-ray diffractograms of the Co_3_O_4_/CeO_2_ samples are presented in Figure 2. All patterns exhibited the same signals previously assigned to the presence of a cubic phase of ceria, with additional signals at 2θ = 19.1, 31.3, 37.1, 45.0, and 65.5° attributable to a cubic spinel phase of Co_3_O_4_ (ICCD 00-042-1467). No signals associated with reduced forms of CeO_2_ or Co_3_O_4_ were detected. The estimation of the crystallite sizes by the Scherrer equation (Table 4) evidenced no change in the crystallite size of CeO_2_ of the supports after cobalt incorporation. On the other hand, the crystallite size of Co_3_O_4_ varied between 26 nm for the Co-DC catalyst and 44 nm for the Co-CC catalyst. It must be noted that the catalysts derived from the CeO_2_ supports with larger specific surface areas (DC, CN, and SCS) consistently presented significantly smaller crystallites.

**Figure 3:**
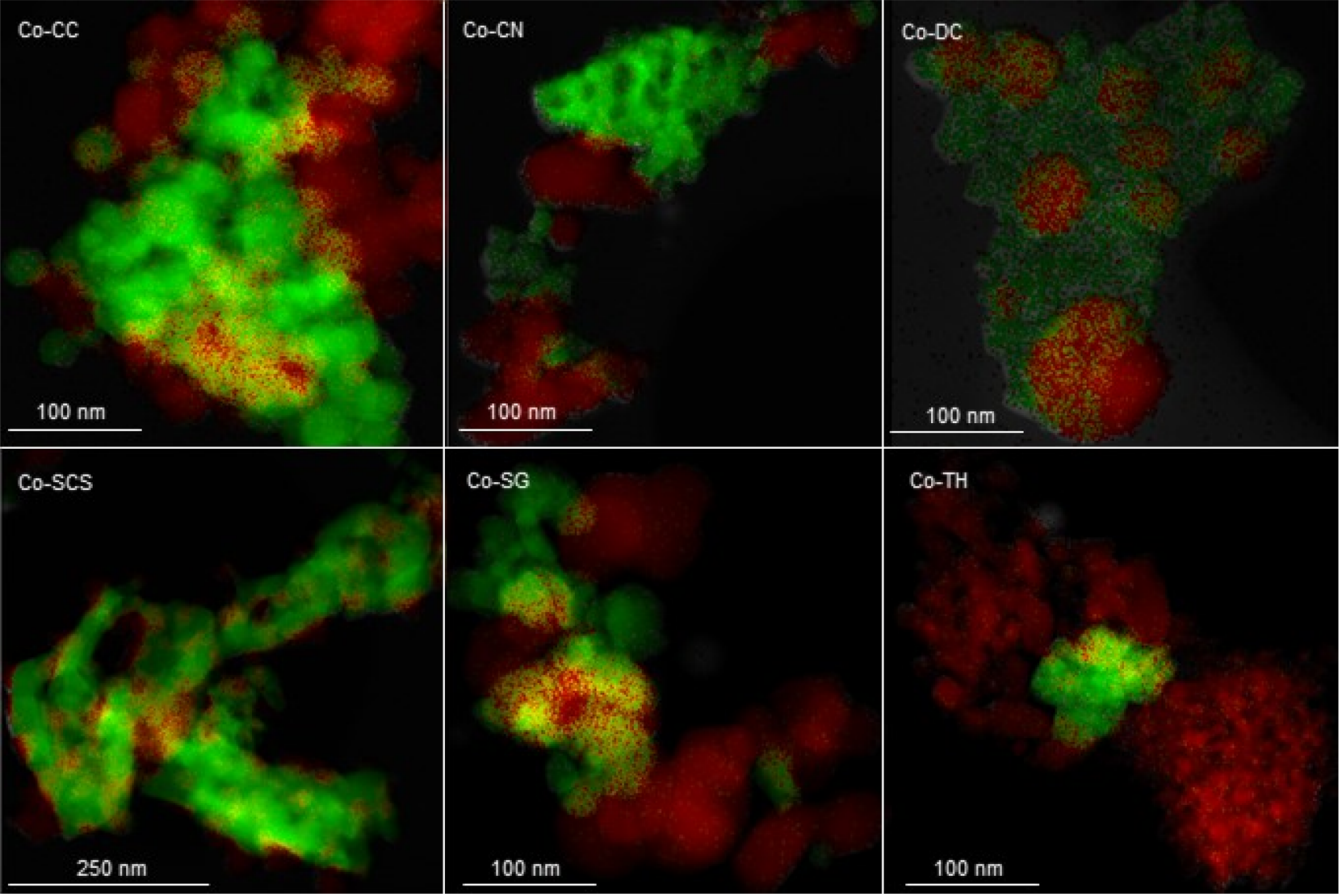
STEM-HAADF/EDS elemental maps of the Co_3_O_4_/CeO_2_ catalysts. Green = Ce and red = Co.

The structure of the cobalt catalysts was also investigated by the elemental mapping of Co and Ce using EDS-HAADF. Selected STEM micrographs are presented in Figure 3. The elemental maps were highly useful for visualizing the aforementioned segregation of Co_3_O_4_ in some of the supported samples. Specifically, the maps of the Co-CC, Co-SG, and Co-TH catalysts presented a significant fraction of Co_3_O_4_ particles completely separated from those of ceria. On the other hand, in the case of the Co-CN, Co-DC, and Co-SCS catalysts, a majority of the cobalt crystallites were located over the surface of the ceria clusters, thus evidencing that they were suitably supported and interacted with the ceria matrix.

**Figure 4:**
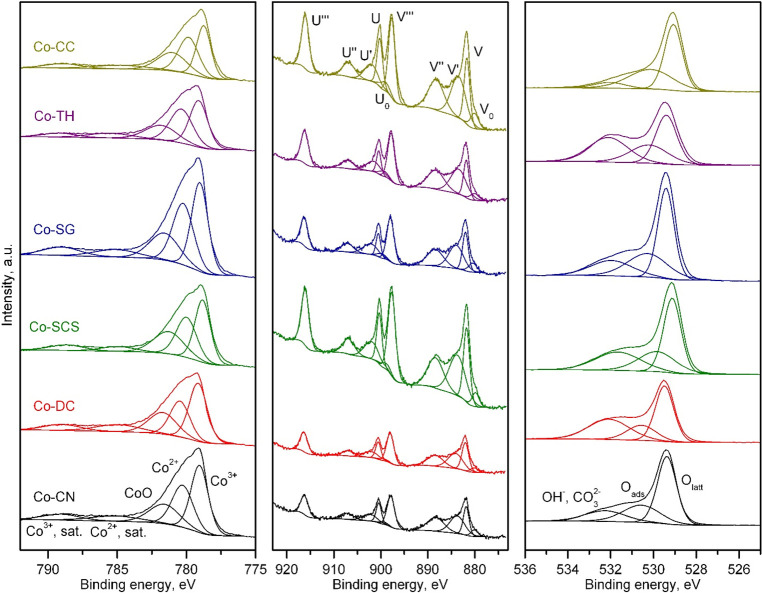
Co 2p (left), Ce 3d (middle), and O 1s (right) XPS spectra of the Co_3_O_4_/CeO_2_ catalysts.

The surface composition of the Co_3_O_4_/CeO_2_ catalysts was also investigated by deconvolution and quantification of the Co 2p_3/2_, Ce 3d, and O 1s XPS spectra (Figure 4). The Ce 3d and O 1s spectra were fitted following the same procedure applied to the blank ceria supports. The Co 2p_3/2_ spectra were deconvoluted into three main signals and two satellite signals. The three main signals, located at 779.2, 780.5, and 782.2 eV, were associated with the presence of Co^3+^ and Co^2+^ in the Co_3_O_4_ lattice and Co^2+^ cations occupying octahedral positions in a CoO-type lattice, respectively.^48^ The presence of CoO on the surface of the cobalt catalysts was probably due to the vacuum conditions of the XPS apparatus. The two satellite signals, centered at 785.0 and 789.1 eV, were related to the Co^2+^ and Co^3+^ ions, respectively.^49^

The surface composition of the cobalt catalysts was also derived from the deconvoluted spectra, and the results are shown in Table 5. First, it must be noticed that the surface composition of all of the catalysts differed significantly from the bulk composition measured by ICP-AES. Thus, the Co/Ce ratio estimated by XPS was higher (1.1–3.4) in comparison to the bulk ratio (around 0.6), which indicated that most of the deposited cobalt was accumulated on the surface of the catalysts. Specifically, Co-DC, Co-CN, and Co-SG presented the highest enrichment of cobalt on the surface. On the other hand, for the Co-CC, Co-SCS, and Co-TH catalysts the Co/Ce ratio was more comparable to that of the bulk, thus indicating that their structure was similar to that of a Co_3_O_4_/CeO_2_ physical mixture, in line with the marked presence of segregated Co_3_O_4_ in these catalysts.

**Table 5:** Surface Composition of the Co_3_O_4_/CeO_2_ Catalysts^a^

catalyst	Ce, wt %	Co, wt %	Co/Ce	Co^3+^/Co^2+^ molar ratio	Ce^4+^/Ce^3+^ molar ratio	O_latt_/O_ads_ molar ratio
Co-CC	32.5	34.4	1.06 (0.58)	1.01	2.15	1.35
Co-CN	14.5	48.4	3.34 (0.62)	1.31	2.28	2.00
Co-DC	14.0	47.5	3.39 (0.63)	1.49	2.46	2.36
Co-SCS	27.5	35.5	1.29 (0.65)	1.20	2.36	1.75
Co-SG	16.2	43.7	2.70 (0.65)	1.14	2.01	1.54
Co-TH	22.2	32.2	1.45 (0.63)	1.09	2.00	1.48

a The values in parentheses are calculated from the ICP-AES results.

The Ce^4+^/Ce^3+^ molar ratio after cobalt addition also depended on the nature of the used ceria support. Hence, the Ce^4+^/Ce^3+^ molar ratio of the Co-CC (2.15), Co-SG (2.01), and Co-TH (2.00) catalysts was comparable to that of their corresponding bare supports (2.19, 1.96, and 2.00, respectively). This was coherent evidence of the low interaction of the cobalt and cerium phases in these three samples. In contrast, the presence of cobalt induced a decrease in the Ce^4+^/Ce^3+^ molar ratio for the Co-DC, Co-CN, and Co-SCS catalysts. This alteration could be a consequence of the notable interaction between cobalt and ceria, thereby resulting in an increase in the oxidation state of cobalt via the electronic equilibrium Ce^4+^ + Co^2+^ ↔ Ce^3+^ + Co^3+^.^50^ A higher Co^3+^/Co^2+^ molar ratio (1.20–1.49) was consistently noticed. Moreover, this seemed to favor the presence of lattice oxygen species on the surfaces of these catalysts.

**Figure 5:**
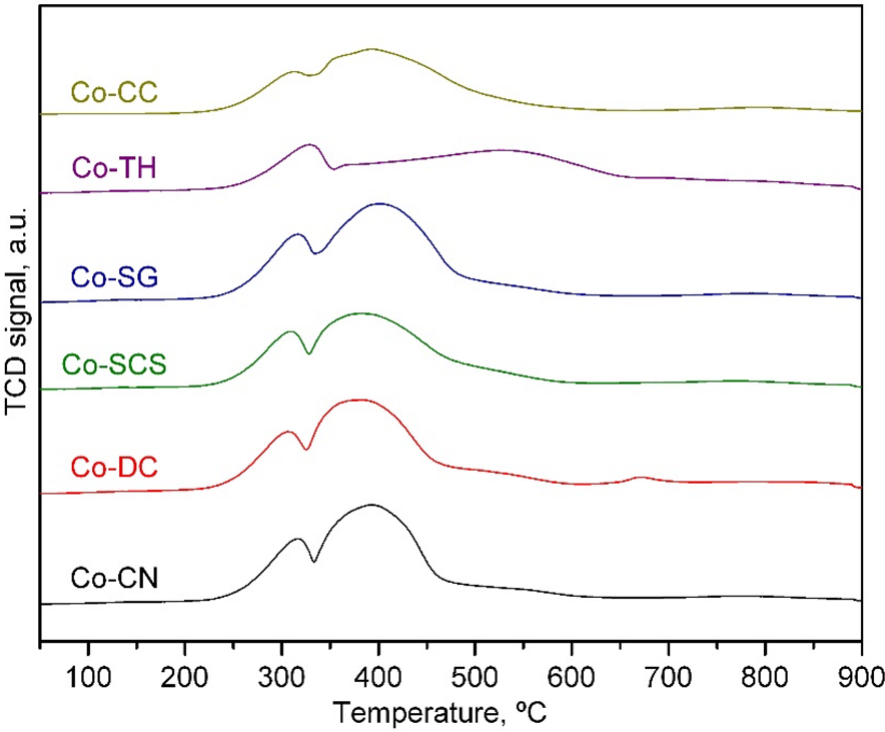
H_2_-TPR profiles of the Co_3_O_4_/CeO_2_ catalysts.

The redox properties of the cobalt catalysts were investigated by H_2_-TPR. All TPR traces (Figure 5) presented three distinctive H_2_ uptakes, two at low temperatures (<600 °C) and another one at high temperatures (>600 °C). The low-temperature contributions corresponded to the reduction of Co_3_O_4_ which took place in two steps: first the Co^3+^ ions were reduced to Co^2+^ at around 250–325 °C, and then the Co^2+^ ions were reduced to metallic cobalt at temperatures of between 375 and 450 °C, depending on the catalyst.^51^ The ceria surface reduction also occurred in this temperature window, but its corresponding H_2_ was much more limited and thus was masked by the reduction of Co_3_O_4_. On the other hand, the high-temperature H_2_ uptake (>600 °C) of the profiles corresponded to the bulk reduction of the CeO_2_ supports. For all synthesized catalysts, the first reduction step peaked at around 300 °C without important differences among the catalysts. The second step, for all of the catalysts except for Co-TH, was located at around 400 °C with a comparable shape and width. For only the Co-TH catalyst, the peak was much wider and its maximum was located at around 530 °C.

The integration and quantification of the TPR profiles, in a similar way to that made for the CeO_2_ supports, allowed the estimation of the degree of reduction of the ceria support since it was assumed that all of the deposited Co_3_O_4_ was completely reduced at temperatures over 600 °C. The results, shown in Table 6, evidenced the highest reducibility of ceria over the Co-CN and Co-DC catalysts, which revealed that the supports of these catalysts possessed a notable amount of highly reducible oxygen species at low temperature.

**Table 6:** H_2_ Uptakes and Degrees of Reduction of the Synthesized Co_3_O_4_/CeO_2_ Catalysts

catalyst	low-temperature H_2_ uptake, mmol g^–1^	total H_2_ uptake, mmol g^–1^	CeO_2_ reduction degree, %	O_2_ consumption at low temperature (CH_4_-TPRe), mmol g_Co_^–1^
Co-CC	6.30	7.20	36	0.14
Co-CN	7.46	7.94	66	0.24
Co-DC	7.49	8.16	76	0.29
Co-SCS	7.01	7.95	57	0.21
Co-SG	7.63	7.84	50	0.17
Co-TH	5.07	7.69	46	0.17

Since the redox behavior of the Co_3_O_4_ phase did not vary significantly, a second effort to gain more insight into the redox properties of the catalysts was made by substituting H_2_ by CH_4_. The respective CH_4_-TPRe profiles that resulted from monitoring the *m*/*z* = 44 mass (CO_2_) are represented in Figure S6, Supporting Information. All traces suggested a two-step process for CH_4_ consumption: a low-temperature step located between 400 and 500 °C attributed to the oxidation of methane by oxygen species associated with Co^3+^ ions and a high-temperature step occurring above 550 °C which corresponded to the oxidation of methane by oxygen species bonded to Co^2+^ ions. Note that under these conditions the formation of CO and H_2_ activated by the in situ-generated metallic cobalt was also detected.

**Figure 6:**
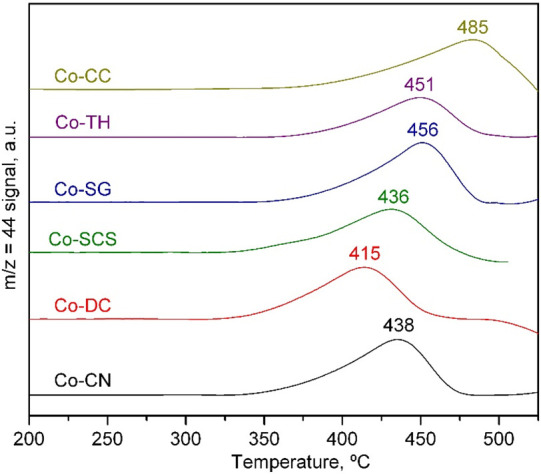
CH_4_-TPRe profiles of the Co_3_O_4_/CeO_2_ catalysts in the 200–550 °C range.

Figure 6 shows a close-up view of the results of this CH_4_-TPRe run between 200 and 550 °C. The higher reactivity of the Co-DC catalyst was clearly evidenced since it showed low-temperature CO_2_ peak production at a temperature of 415 °C, 20 °C lower with respect to the rest of the examined catalysts. The integration of these low-temperature events allowed for the calculation of the quantity of active oxygen species, expressed as the amount of O_2_ consumed in the complete oxidation of methane present in each sample. The results are shown in Table 6. Thus, the O_2_ consumption of the catalysts varied from 0.14 mmol g_Co_^–1^ for the Co-CC catalyst to 0.29 mmol g_Co_^–1^ for the Co-DC catalyst. This sample, along with the Co-CN and Co-SCS catalysts, contained the largest amount of active oxygen species.

### 3.3 Catalytic Performance of the Co_3_O_4_/CeO_2_ Catalysts

**Figure 7:**
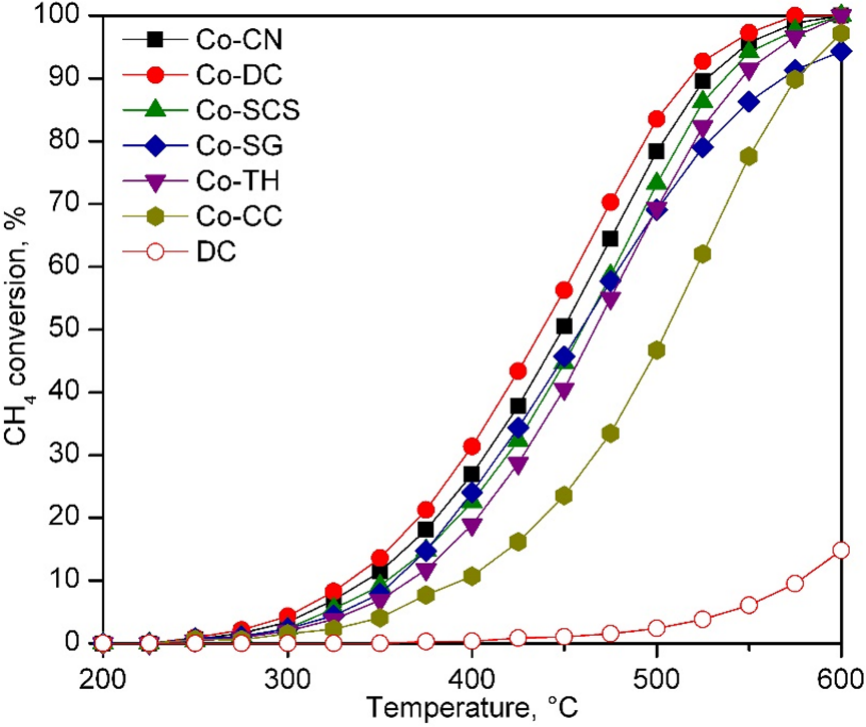
Light-off curves of the Co_3_O_4_/CeO_2_ catalysts.

The efficiency of the synthesized Co_3_O_4_/CeO_2_ catalysts was evaluated by obtaining their respective light-off curves at 60 000 h^–1^. These light-off curves were obtained between 200 and 600 °C. For each catalyst, three consecutive tests were carried out, and the results from the last cycle were taken as the representative conversion–temperature profile for a given catalyst (Figure 7) since, in all cases, the second and third were identical. To ensure that the obtained kinetic results were not affected by mass- or heat-transfer limitations, the criteria for intraparticle and extraparticle mass and energy diffusion, as well as the temperature gradients, were checked (Table S1, Supporting Information).

First, it must be noticed that all catalysts achieved 100% selectivity for CO_2_. By direct inspection of the light-off curves, it could be clearly concluded that the Co-CC catalyst presented the worst behavior among the investigated catalysts while the Co-DC catalyst presented the best efficiency. The *T*_50_ values extracted from the light-off profiles (Table 7) established the following conversion trend: Co-DC > Co-CN > Co-SCS > Co-SG > Co-TH > Co-CC. This trend was coherent with the main findings derived from the characterization results since the most suitable catalysts were those supported over the ceria supports with better textural and structural properties (DC, CN, and SCS). Also, these catalysts presented the highest reducibility and population of active oxygen species. For comparative purposes, the light-off curve of the bare DC ceria support is included in Figure 7. As can be observed, its efficiency was rather poor, with only a 15% conversion at 600 °C. Additionally, instead of CO_2_, methane was selectively converted to CO.

**Table 7:** Kinetic Parameters of the Synthesized Co_3_O_4_/CeO_2_ Catalysts

catalyst	*T*_50_, °C	reaction rate @ 375 °C, mmol CH_4_ h^–1^ g_CAT_^–1^	activation energy, kJ mol^–1^
Co-CC	500	0.62	82
Co-CN	450	1.40	77
Co-DC	435	1.68	74
Co-SCS	455	1.13	74
Co-SG	460	0.98	79
Co-TH	470	0.85	83

The specific reaction rate of the cobalt catalysts at 375 °C was estimated by the differential method (methane conversion <20%). The obtained values followed the same aforementioned trend, with the most active catalyst being the Co-DC sample (1.68 mmol CH_4_ h^–1^ g_CAT_^–1^) and the least active being the Co-CC catalyst (0.62 mmol CH_4_ h^–1^ g_CAT_^–1^) (Table 7). Likewise, the apparent activation energies were calculated from the light-off curves by the integral method, assuming zeroth order for the oxygen and first order for methane (Figure S7, Supporting Information), which are reaction orders comparable to those observed for the methane combustion reaction under excess oxygen.^13,52^ The resulting activation energies were in the 74–82 kJ mol^–1^ range, in fairly good agreement with those reported in the literature for this reaction over Co_3_O_4_-based catalysts.^53,54^

**Figure 8:**
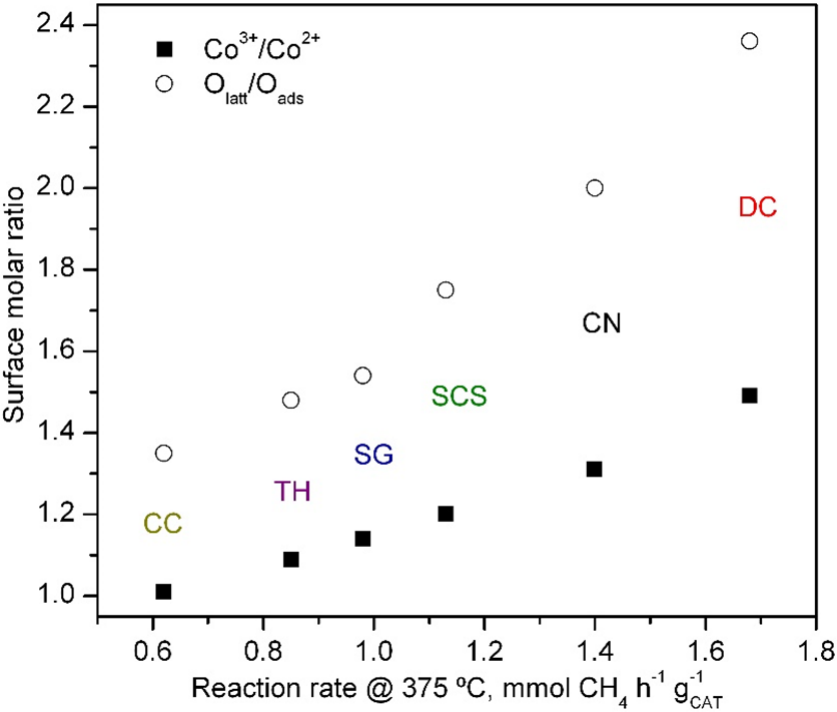
Correlation between the activity of the Co_3_O_4_/CeO_2_ catalysts and their surface composition.

**Figure 9:**
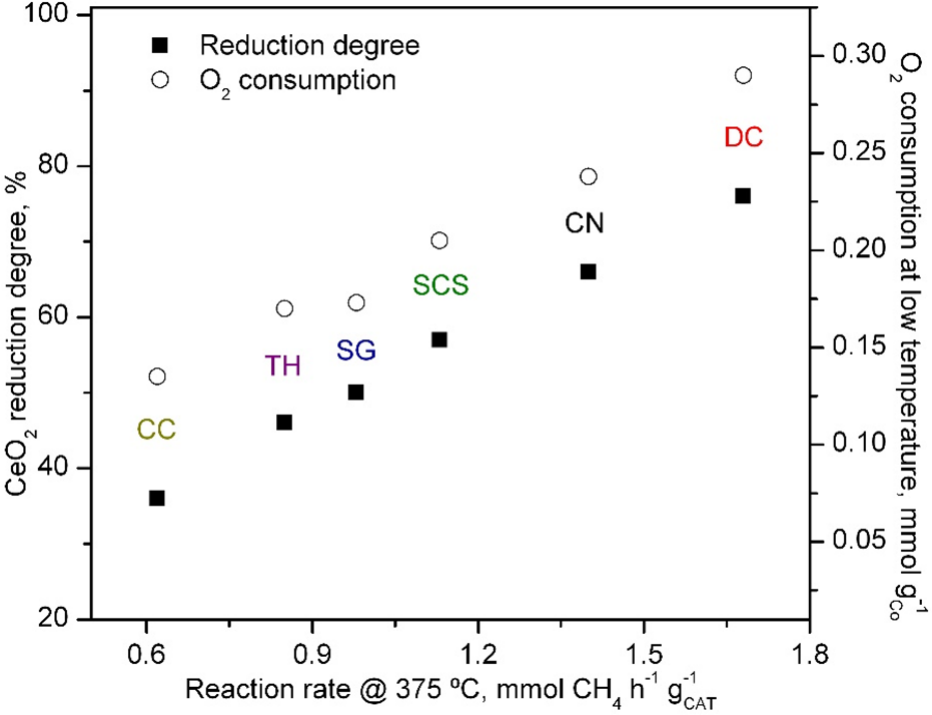
Correlation between the activity of the Co_3_O_4_/CeO_2_ catalysts and their redox properties.

The remarkable intrinsic activity of the Co-CN and Co-DC catalysts seemed to be directly related to the better textural properties of the respective CeO_2_ support as well as to its higher oxidation state of cerium species (Ce^4+^/Ce^3+^ molar ratio) at the surface. This led to a more intense promotion of the redox properties of the deposited Co_3_O_4_. This beneficial influence was clearly observed by the good relationship among the Ce^4+^/Ce^3+^, Co^3+^/Co^2+^, and O_latt_/O_ads_ molar ratios obtained from XPS, as dictated by Figure 8. In addition, the strong correlation between the degree of reduction of CeO_2_ in the cobalt catalysts (H_2_-TPR) and the O_2_ consumption at low temperatures (CH_4_-TPRe) with the specific reaction rate (Figure 9) evidenced that the aforementioned redox promotional effect increased the content of active oxygen species in the lattice of the cobalt oxide present in the Co-DC and Co-CN catalysts. This was ultimately the reason for the observed higher catalytic activity of these two Co_3_O_4_/CeO_2_ samples.

**Figure 10:**
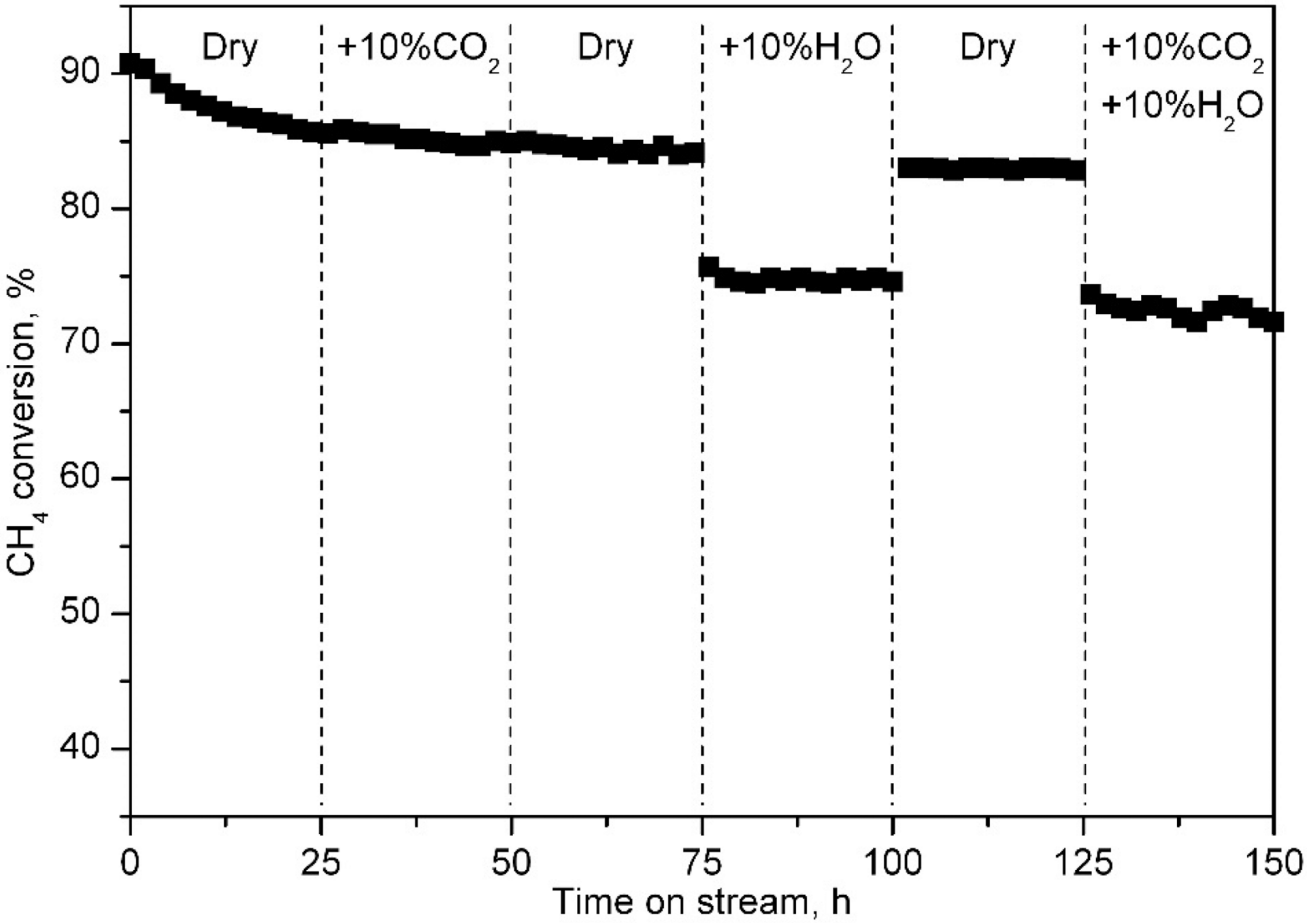
Evolution of the methane conversion with time on stream over the Co-DC catalyst at 525 °C.

Finally, both the thermal and hydrothermal stabilities of the most active catalyst (Co-DC) were studied at constant temperature (525 °C, 60 000 h^–1^) under several consecutive combinations of reaction conditions for a total time on stream of 150 h. Figure 10 shows the evolution of the methane conversion under these conditions.

The initial methane conversion was around 90%, a value comparable to that obtained in the light-off experiment at 525 °C, but rapidly decreased to 85% over the first 25 h under dry conditions. The addition of 10 vol % CO_2_ to the feedstream did not seem to have any negative effect since the conversion remained stable at 85% after 75 h of experiment. Once the admission of water (10 vol %) started, the conversion level appreciably decreased to 75% until the water was turned off again. Then, the conversion recovered almost completely (84%). This good behavior, compared to that of Co_3_O_4_ catalysts supported over other porous supports, such as γ-Al_2_O_3_,^55^ could be explained by the more hydrophobic character of CeO_2_, which probably inhibited water adsorption to a larger extent.^56^ Finally, the coaddition of water vapor and CO_2_ during the last 25 h of time on stream did not seem to have any additional detrimental effect on conversion, apart from that already described due to the presence of water vapor.

The almost complete recovery of the conversion value (from 90 to 85%) after water vapor was no longer supplied to the reactor pointed out that inhibition by water vapor was almost completely reversible and was due to only the physical adsorption of water molecules on the active Co_3_O_4_ crystallites. However, the state of the used sample after the stability test was extensively analyzed. Thus, the specific surface area of the used catalyst was 49 m^2^ g^–1^ while the Co_3_O_4_ crystallite size was 25 nm, which indicated that no sintering of the catalyst occurred during the stability tests. Furthermore, the CH_4_-TPRe profile of the used catalyst (Figure S8, Supporting Information) was essentially identical to that of the fresh catalyst, and the corresponding O_2_ consumption at low temperature was similar, which suggested that the exposition to water vapor did not induce any permanent effect on the redox properties of the Co-DC catalyst. Finally, attention was paid to examining the surface composition of the sample by XPS. The XPS spectra (Co 2p, Ce 3d, and O 1s) are included in Figure S9, Supporting Information. A certain increase in the amount of reduced species (Co^2+^ and Ce^3+^) was observed. Thus, the Co^3+^/Co^2+^ (1.42) and Ce^4+^/Ce^3+^ (2.30) molar ratios slightly decreased with respect to those of the fresh sample (1.49 and 2.46, respectively). As a consequence, the used sample contained a lower relative abundance of active lattice oxygen species as evidenced by the lower O_latt_/O_ads_ molar ratio (2.04; 2.36 for the fresh sample).

## 4 Conclusions

Six supported Co_3_O_4_/CeO_2_ catalysts were synthesized by a basic precipitation method and examined for the complete oxidation of methane. The catalytic supports were prepared by various synthesis routes, and the effect of the physicochemical properties of the CeO_2_ supports on the catalytic behavior of the cobalt catalysts was investigated.

The six examined synthesis routes were precipitation with ammonia (CN), direct calcination of cerium nitrate (DC), solution combustion synthesis (SCS), sol–gel complexation (SG), precipitation assisted by a surfactant (TH), and basic precipitation with sodium carbonate (CC). Among the synthesized CeO_2_ supports, those obtained by the CN and DC methodologies exhibited the best textural and structural properties, with specific surface areas of around 60 m^2^ g^–1^. Moreover, the structural characterization of the cobalt catalysts supported on the other four supports demonstrated that those presented various degrees of Co_3_O_4_ segregation due to not having enough surface area to deposit all of the cobalt that was incorporated.

The analysis of the surface composition and the redox properties of the ceria supports and the cobalt catalysts revealed that the Co-DC and Co-CN catalysts were supported over ceria supports with a significantly higher content of Ce^4+^ ions, that is, with a higher degree of oxidation. As a consequence, these two catalysts exhibited a favored presence of Co^3+^ ions on their surfaces, which led to a higher concentration of active lattice oxygen species. For these reasons, these two catalysts were found to be the most active. In addition, owing to the highly hydrophobic character of CeO_2_, the best catalyst of the set (Co-DC) showed a notable resistance to deactivation by the presence of water vapor. Thus, in the presence of 10 vol % water vapor it suffered only a slight loss of conversion, which turned out to be completely reversible and did not have any irreversible effect on the physicochemical properties of the catalyst.

A global contextualization of the reported results in this study points out that although our Co_3_O_4_/CeO_2_ optimized catalyst exhibits reasonably good behavior under dry conditions, the sample does not meet the requirement of attaining >90% conversion under realistic conditions for natural gas-fueled vehicles, including a typical temperature for exhaust gases below 550 °C and the notable presence of water vapor. One possible strategy for appreciably enhancing the catalytic efficiency of Co_3_O_4_-based catalysts could be the addition of controlled amounts of Pd (<1 wt %) in spite of the fact that this would somewhat increase the cost of the resultant catalyst. An alternative proposal to be considered could be the use of Ce-modified supports with structural dopants such as Zr and Pr. The insertion of these cations into the lattice of the ceria markedly improves the intrinsic redox properties of the Ce/Zr and Ce/Pr mixed oxides, thus resulting in attractive supports for oxidation catalysts.

## Supporting Information Available

Criteria for the accurate analysis of intrinsic reaction rates of the foam catalysts; N_2_ physisorption isotherms of the CeO_2_ supports; pore size distributions of the CeO_2_ supports and Co_3_O_4_/CeO_2_ catalysts; XRD patterns of the CeO_2_ supports; Ce 3d and O 1s XPS spectra of the CeO_2_ supports; N_2_ physisorption isotherms of the Co_3_O_4_/CeO_2_ catalysts; CH_4_-TPRe profiles of the Co_3_O_4_/CeO_2_ catalysts; Arrhenius plots of the Co_3_O_4_/CeO_2_ catalysts; CH_4_-TPRe profiles of the Co-DC sample before and after the stability test in the 200–500 °C range; and Co 2p, Ce 3d, and O 1s XPS spectra of the Co-DC catalyst before and after the stability tests (PDF)
