# Supplementary material for: On the Effect of the Synthesis Route of the Support in Co3O4/CeO2 Catalysts for the Complete Oxidation of Methane
Source: Ind Eng Chem Res. 2022 Dec 6;61(49):17854–65. doi: 10.1021/acs.iecr.2c03245 (PMC9756389; doi:10.1021/acs.iecr.2c03245)
Supplement: Supplementary file 1 — ie2c03245_si_001.pdf [file ie2c03245_si_001.pdf]

SUPPORTING INFORMATION

On the effect of the synthesis route of the support in Co_3O_4/CeO_2 catalysts for complete oxidation of methane

Andoni Choya[1,*], Beatriz de Rivas[1], Jose Ignacio Gutiérrez-Ortiz[1] and Rubén López-Fonseca[1]

[1]Chemical Technologies for Environmental Sustainability Group, Department of Chemical Engineering, Faculty of Science and Technology, University of The Basque Country UPV/EHU, Barrio Sarriena s/n, Leioa, Bizkaia E-48940, Spain

*Corresponding author:

Phone: +34-94-6013485

Fax: +34-94-6015963

E-mail address: andoni.choya@ehu.eus

Table S1. Criteria for accurate analysis of intrinsic reaction rates of the foam catalysts (as estimated for the Co-DC catalyst at 375 °C).

Figure S1. N_2 physisorption isotherms of the CeO_2 supports.

Figure S2. Pore size distributions of the CeO_2 supports and Co_3O_4/CeO_2 catalysts.

Figure S3. XRD patterns of the CeO_2 supports.

Figure S4. Ce3d (left) and O1s (right) XPS spectra of the CeO_2 supports.

Figure S5. N_2 physisorption isotherms of the Co_3O_4/CeO_2 catalysts.

Figure S6. CH_4-TPRe profiles of the Co_3O_4/CeO_2 catalysts.

Figure S7. Arrhenius plots of the Co_3O_4/CeO_2 catalysts.

Figure S8. CH_4-TPRe profiles of the Co-DC sample before and after the stability test in the 200-500 °C temperature range.

Figure S9. Co2p (left), Ce3d (centre) and O1s (right) XPS spectra of the Co-DC catalyst before and after the stability tests.

Criteria	Recommendation	At 375 ºC	Least favourable conditions
Minimum bed length	Bodenstein parameter < $L_{bed}/d_{particle}$	4.5 < 79.5	55.6 < 79.5[b]
Minimum reactor diameter	$d_{tube}/d_{particle} > 10$	31.2[a]	31.2[a]
Extraparticle mass transfer	Carberry number < 0.05/n	0.0042	0.046[b]
Extraparticle heat transfer	$\gamma\beta_e Ca < 0.05$	0.0017	0.028[c]
Intraparticle mass transport	Wheeler-Weisz modulus < 0.15	$4.3\cdot10^{-4}$	$8.0\cdot10^{-4}$[d]
Intraparticle heat transport	Mears parameter < 0.1	$2.0\cdot10^{-6}$	$4.2\cdot10^{-6}$[d]
Radial temperature gradient	Radial gradient < 0.05	0.004	0.0057[d]
Intraparticle temperature gradient	Temperature gradient < 0.3	0.15	0.175[c]
Bed dilution	Bed dilution parameter < 0.05	0.047	0.047

[a]The value of this ratio is 25.4 if the thermocouple is taken into consideration for calculation of reactor diameter.
[b]Determined at 500 ºC.
[c]Determined at 600 ºC.
[d]Determined at 450 ºC.

For the definition of each parameter the readers should refer to Eurokin - http://eurokin.org/; J. Perez-Ramirez, R.J. Berger, G. Mul, F. Kapteijn, J.A. Moulijn, Catalysis Today 60 (2000) 93-109 or A. Aranzabal, J.A. González-Marcos, J.L. Ayastuy, J.R. González-Velasco, Chem. Eng. Sci. 61 (2006) 3564-3576.

TABLE S1

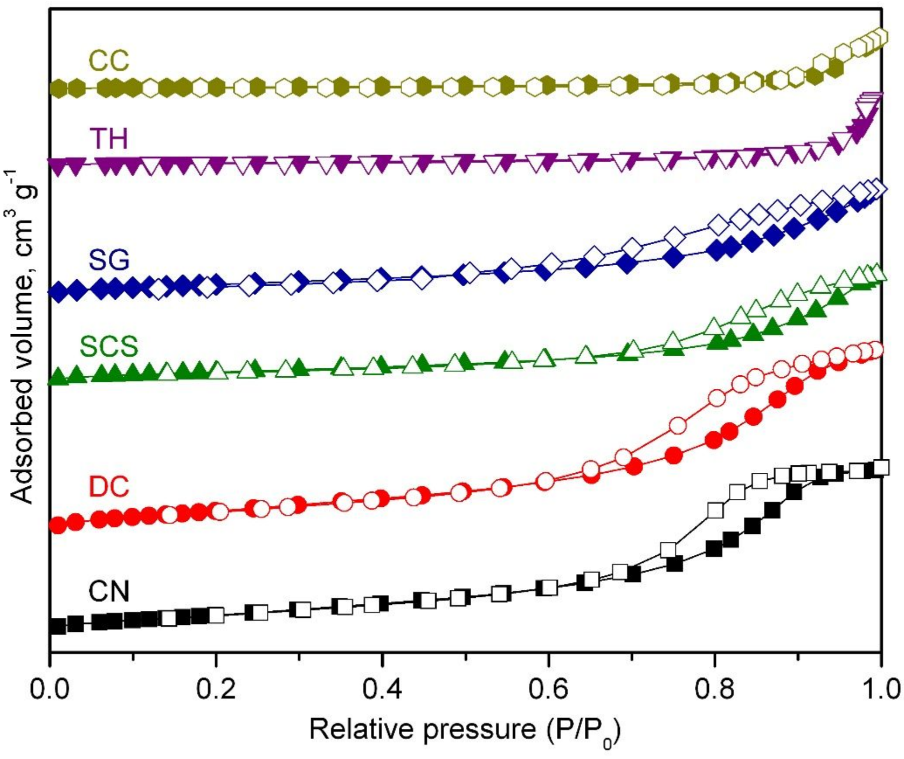
CC
TH
SG
SCS
DC
CN
Adsorbed volume, cm^3 g^{-1}
0.0
0.2
0.4
0.6
0.8
1.0
Relative pressure (P/P$_0$)

FIGURE S1

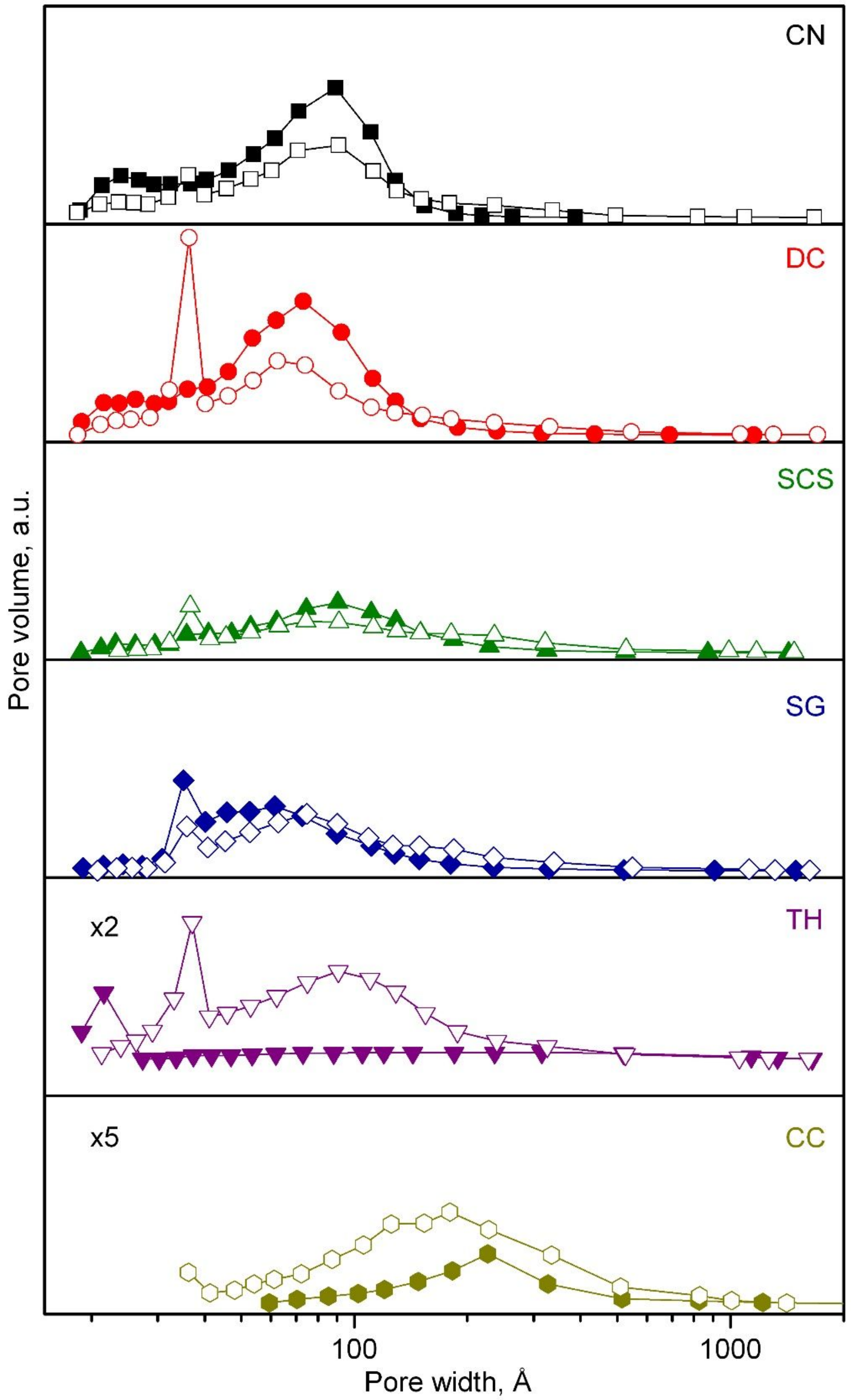

FIGURE S2

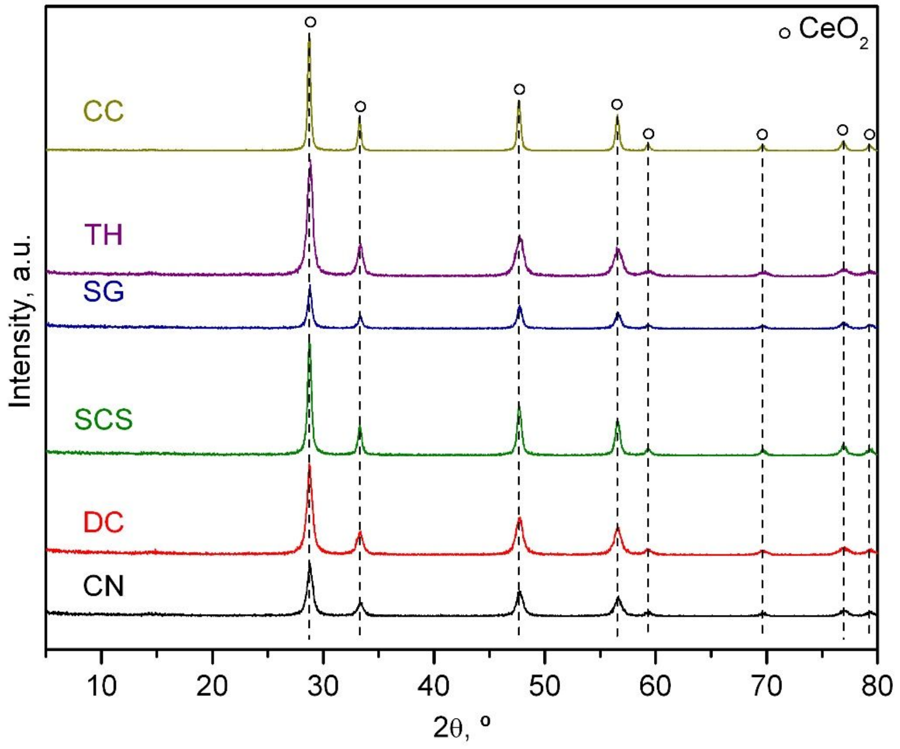

FIGURE S3

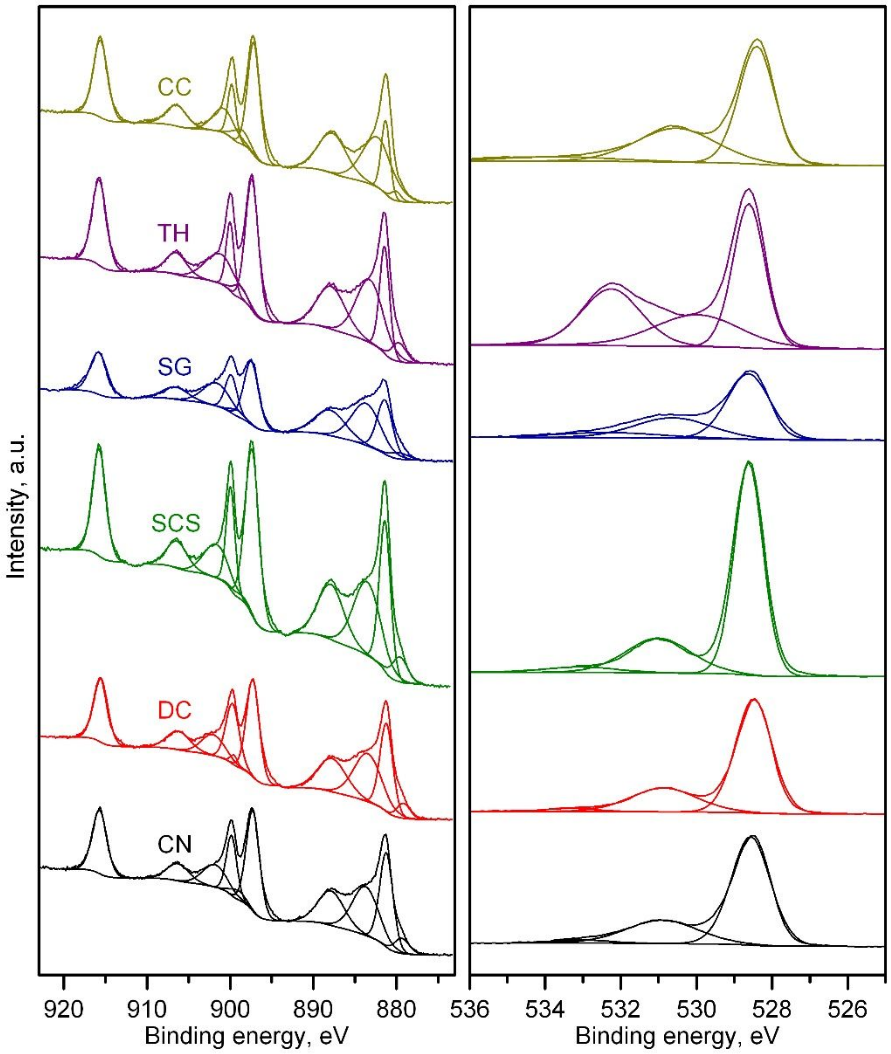
CC
TH
SG
SCS
DC
CN
Intensity, a.u.
920
910
900
890
880
Binding energy, eV
536
534
532
530
528
526
Binding energy, eV

FIGURE S4

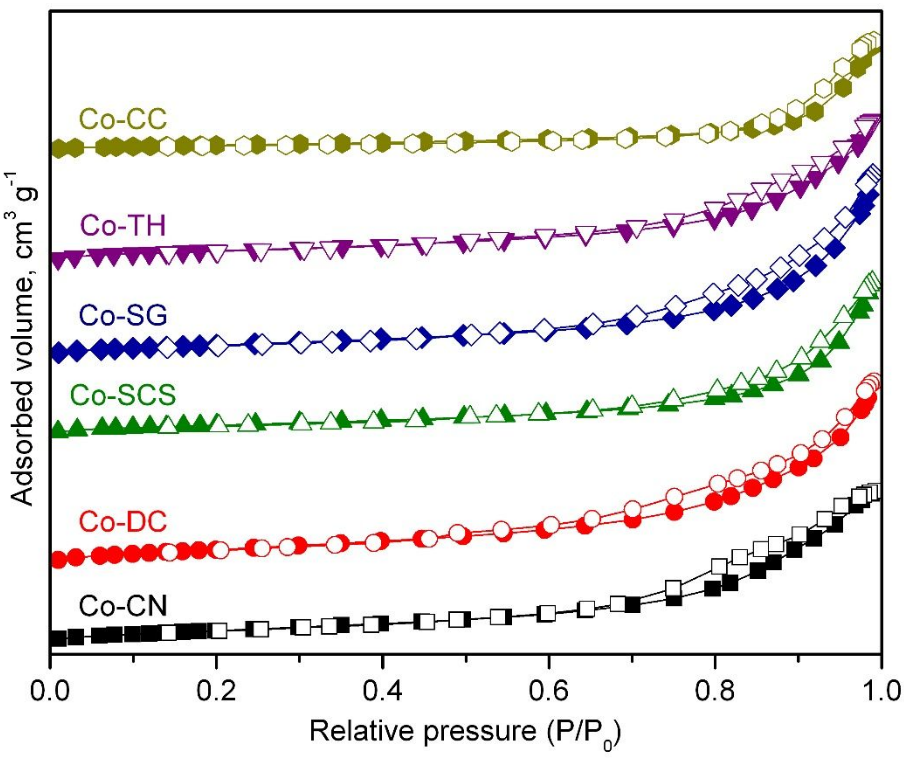

Co-CC
Co-TH
Co-SG
Co-SCS
Co-DC
Co-CN
Adsorbed volume, cm^3 g^{-1}
0.0
0.2
0.4
0.6
0.8
1.0
Relative pressure (P/P$_0$)

FIGURE S5

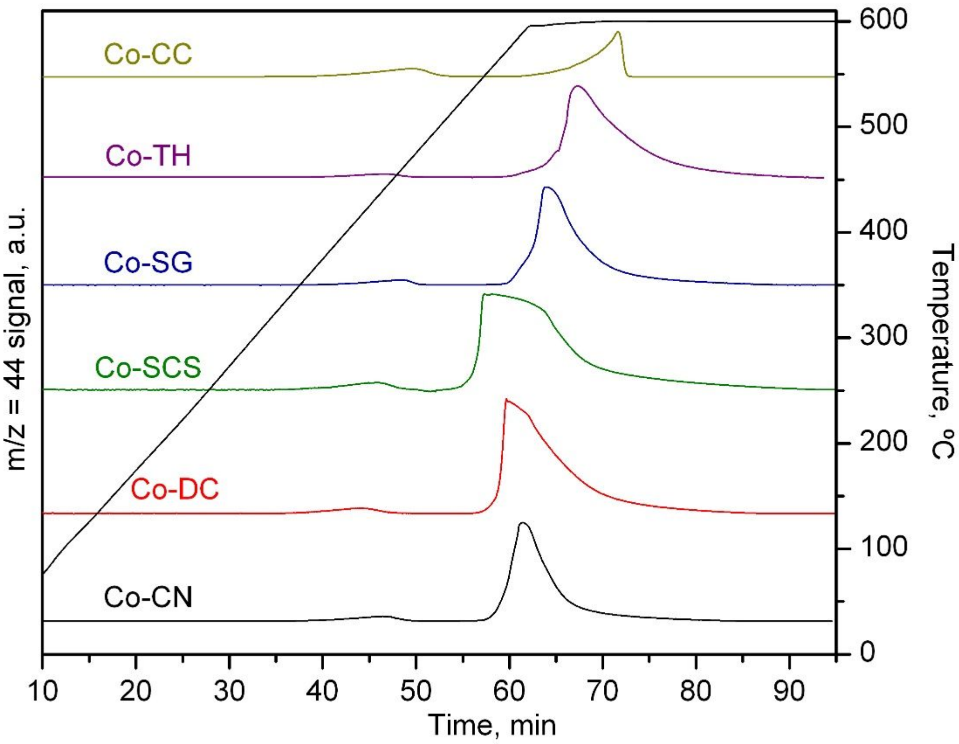

Co-CC
Co-TH
Co-SG
Co-SCS
Co-DC
Co-CN
m/z = 44 signal, a.u.
Temperature, °C
600
500
400
300
200
100
0
10
20
30
40
50
60
70
80
90
Time, min

FIGURE S6

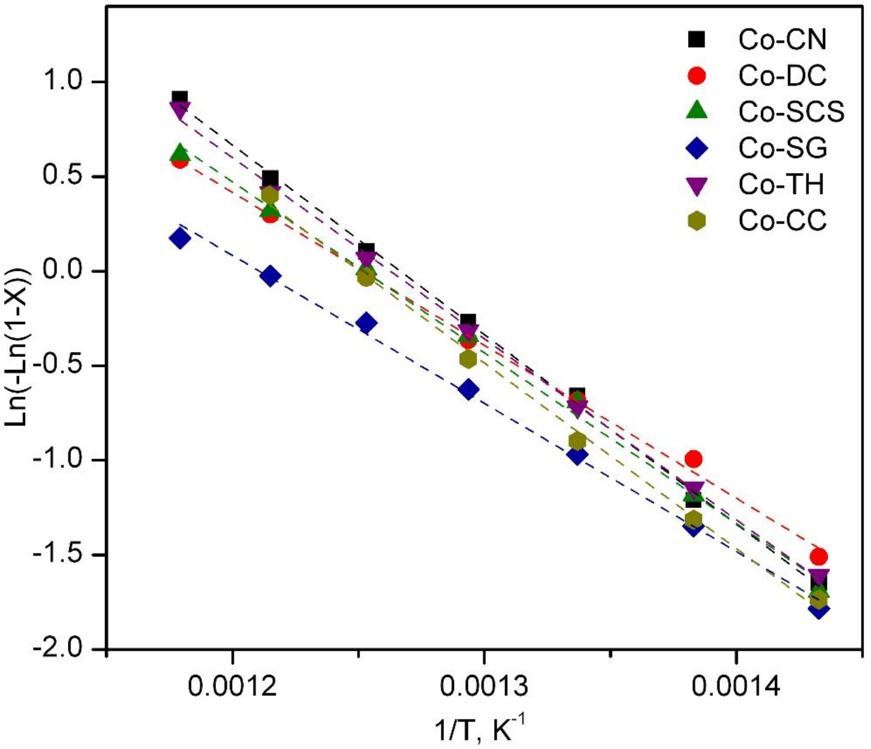

Co-CN
Co-DC
Co-SCS
Co-SG
Co-TH
Co-CC
1.0
0.5
0.0
-0.5
-1.0
-1.5
-2.0
Ln(-Ln(1-X))
0.0012
0.0013
0.0014
1/T, K-1

FIGURE S7

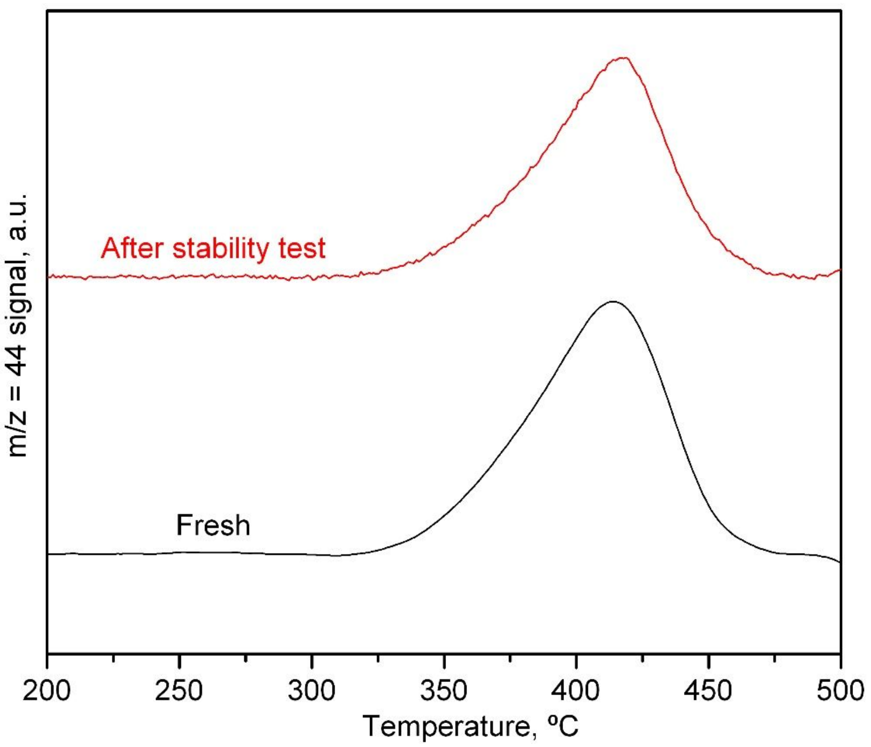
After stability test
Fresh
m/z = 44 signal, a.u.
200
250
300
350
400
450
500
Temperature, ºC

FIGURE S8

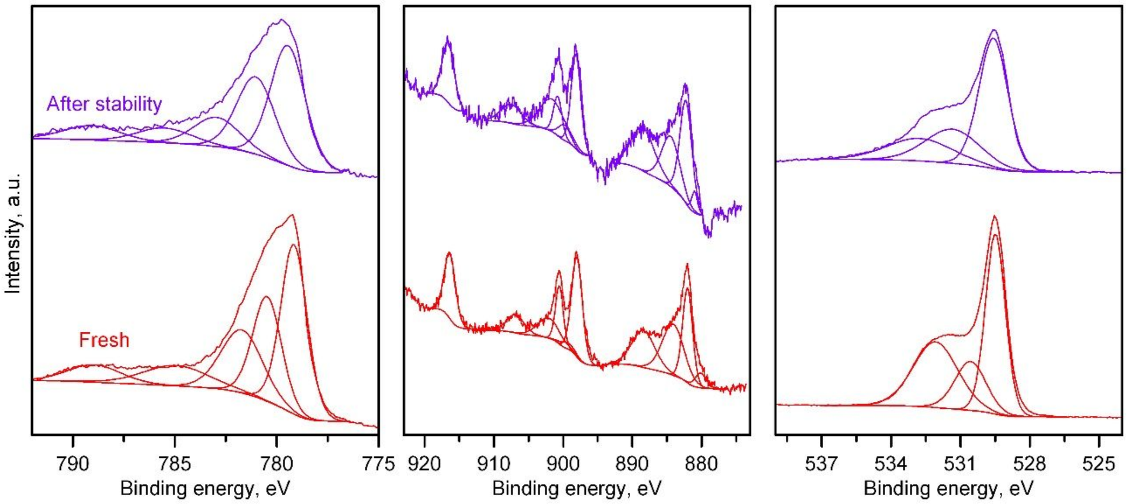
After stability
Fresh
Intensity, a.u.
790
785
780
775
Binding energy, eV
920
910
900
890
880
Binding energy, eV
537
534
531
528
525
Binding energy, eV

FIGURE S9
